# Chlamydial clinical isolates show subtle differences in persistence phenotypes and growth *in vitro*

**DOI:** 10.1099/acmi.0.000204

**Published:** 2021-02-19

**Authors:** Mark Thomas, Amba Lawrence, Samuel Kroon, Lenka A. Vodstrcil, Samuel Phillips, Jane S. Hocking, Peter Timms, Wilhelmina M. Huston

**Affiliations:** ^1^​ School of Life Sciences, Faculty of Science, University of Technology Sydney, Ultimo, NSW, Australia; ^2^​ Institute of Health and Biomedical Innovation, Faculty of Health, Queensland University of Technology, Kelvin Grove, Queensland, Australia; ^3^​ Central Clinical School, Monash University, Melbourne, Victoria, Australia; ^4^​ Melbourne Sexual Health Centre, Alfred Hospital, Carlton, Victoria, Australia; ^5^​ Centre for Epidemiology and Biostatistics, Melbourne School of Population and Global Health, University of Melbourne, Parkville, Victoria, Australia; ^6^​ Murdoch Childrens Research Institute, Parkville 3052, Victoria, Australia; ^7^​ Faculty of Science, Health, Education and Engineering, University of the Sunshine Coast, Sippy Downs, Queensland, Australia

**Keywords:** *Chlamydia trachomatis*, persistence, clinical isolates, iron, penicillin

## Abstract

Urogenital *Chlamydia trachomatis* infection is the most common sexually transmitted bacterial infection throughout the world. While progress has been made to better understand how type strains develop and respond to environmental stress *in vitro*, very few studies have examined how clinical isolates behave under similar conditions. Here, we examined the development and persistence phenotypes of several clinical isolates, to determine how similar they are to each other, and the type strain *C. trachomatis* D/UW-3/Cx. The type strain was shown to produce infectious progeny at a higher magnitude than each of the clinical isolates, in each of the six tested cell lines. All chlamydial strains produced the highest number of infectious progeny at 44 h post-infection in the McCoy B murine fibroblast cell line, yet showed higher levels of infectivity in the MCF-7 human epithelial cell line. The clinical isolates were shown to be more susceptible than the type strain to the effects of penicillin and iron deprivation persistence models in the MCF-7 cell line. While subtle differences between clinical isolates were observed throughout the experiments conducted, no significant differences were identified. This study reinforces the importance of examining clinical isolates when trying to relate *in vitro* data to clinical outcomes, as well as the importance of considering the adaptations many type strains have to being cultured *in vitro*.

## Introduction

*Chlamydia trachomatis* is an obligate intracellular pathogen and is the most common bacterial sexually transmitted infection (STI) worldwide. There are more than 83000 *Chlamydia* infections recorded in Australia each year [1]. The pathology and sequelae associated with chlamydial diseases are thought to be associated with the infected individual’s inflammatory response and potentially influenced by a number of important host factors [2]. In women, chlamydial disease ranges from mild cases of cervicitis, endometritis and salpingitis, to more serious cases of pelvic inflammatory disease (PID), tubal infertility and life-threatening ectopic pregnancy [3–5].

Cases of urogenital *Chlamydia* are treated with either azithromycin or doxycycline. While evidence suggests doxycycline (usually 7 day regimen) is slightly more effective at clearing infection, 1 g single-dose azithromycin is often prescribed for its simple one-off treatment dose [6, 7]. Azithromycin is a broad-spectrum macrolide antibiotic with a relatively long half-life (40–68 h) and high lipid solubility, and accumulates within macrophages migrating to the site of infection [8, 9]. Despite its high efficacy, instances do occur in which monitored women remain infected after treatment. The reasons for this remain unclear, but potential explanations include: reinfection from an untreated partner, chlamydial gastrointestinal colonization and auto-inoculation of the cervical site or treatment failure [10, 11]. Direct macrolide resistance is generally not considered a probable cause due to the antibiotic’s mechanism of action, and the scarcity of clinical isolates with validated genotypic or phenotypic resistance to azithromycin [12–16]. Furthermore, mutants created *in vitro* with single nucleotide polymorphisms (SNPs) in their 23S rRNA and L4 protein-encoding genes show poor biological fitness and viability [17–20], further supporting that their emergence in the population is likely to be rare.

When combined, *in vitro* and clinical evidence suggests that *C. trachomatis* rarely survives treatment, which prpbably reflects both the pathogen and its intracellular niche. One candidate mechanism is chlamydial persistence, which is believed to be a positive and beneficial adaptation unique to the genus *Chlamydia* [21, 22]. The persistence phenotype is characterized by several reversible morphological, transcriptional and metabolic changes [23–27]. The collective profiles of these changes vary depending on the inducer of persistence; however, the morphologically aberrant chlamydial cells observed during persistence, altered inclusion sizes, and the reversible loss of both cultivability and replicative capacity are the universal hallmarks of chlamydial persistence (reviewed previously [28]).

Several environmental conditions and exogenous stimuli have been demonstrated to induce chlamydial persistence *in vitro* [29]. Penicillin and IFN-γ are two of the most extensively studied of these stimuli, and have both been used to characterize different aspects of the persistence phenotype, as reviewed by Wyrick [28]. Use of the iron-chelating agent deferoxamine mesylate has shown that iron restriction not only induces persistence in *C. trachomatis *, but also alters the pathogen’s signalling pathways that modulate host-cell apoptosis [30, 31]. Interestingly, IFN-γ also decreases cellular levels of iron in infected cells by downregulating their transferrin receptor expression [32].

Previous investigations into chlamydial persistence have established that type strains have variations in their degree of responsiveness to certain stimuli, and have altered susceptibilities to antibiotics while in the persistent state [33–36]. In the present study, we examined the *in vitro* phenotypes of selected clinical isolates of *C. trachomatis* isolated during the Australian *Chlamydia* Treatment Study (ACTS) [11]. Their relative abilities to infect and develop in different cell lines were measured, as were their responses to two widely used and physiologically relevant models of *in vitro* chlamydial persistence. Their susceptibility to azithromycin during persistence was also investigated, to better understand whether persistence has implications for treatment with frontline antibiotics when analysed on recent clinical isolates.

## Methods

### Cell culture and cultivation of *Chlamydia*

McCoy B, MCF-7, CACO-2, HeLa, SiHa and ARPE-19 cell lines (details in Table 1) were cultured in Dulbecco’s modified Eagle medium (DMEM) supplemented with 10 % (v/v) fetal calf serum (FCS; Sigma), 4 mM analyl-glutamine (Sigma), 100 μg ml^−1^ streptomycin (Life Technologies) and 50 μg ml^−1^ gentamicin (Life Technologies). A range of cell types available to the study were selected to profile any possible differences in the clinical isolates. All cell lines were incubated at 37 °C in a humid environment containing 5 % CO_2_. All cell lines were regularly confirmed to be free of *Mycoplasma* contamination using either an in-house PCR, or the Mycoalert Plus Mycoplasma Detection Kit assay (Lonza). All six clinical isolates used were obtained from the ACTS. This was a cohort study of women diagnosed with and treated for genital *Chlamydia* [11] to examine factors associated with repeat chlamydia infection. In one case, two isolates from one participant after a repeat positive event were included in the study in the event that they prove to have some detectable difference in persistence phenotypes to those analysed here. Isolates were collected from infected women using swabs stored in a 2 ml cryovial tube containing a sucrose-phosphate glutamate (SPG) buffer, at −80 °C, and couriered on dry ice. A unique code has been generated to identify each isolate purely for the purposes of this paper.

**Table 1.** Mammalian cell lines used in this study

Name	Cell type	Origin	ATCC code
McCoy B	Fibroblast	Fibroblast from *Mus musculus*	CRL-1696
MCF-7	Epithelial	Human mammary epithelium	HTB-22
CACO-2	Epithelial	Human colorectal adenocarcinoma	HTB-37
HeLa	Epithelial	Human cervical carcinoma	CRM-CCL-2
SiHa	Epithelial	Human cervical squamous cell carcinoma	HTB-35
ARPE-19	Epithelial	Human adult retinal pigmented epithelium	CRL-2302

Isolates were cultured from the original cervical swabs from women using a series of culture steps to attain sufficient culture for these experiments (six to 12 passages depending on the isolate). Stocks were stored and propagated from SPG buffer which consisted of 5 mM glutamic acid, 10 mM sodium phosphate and 250 mM sucrose balanced to pH 7.4. The five clinical isolates used in this study are detailed in Table 2.

**Table 2.** Identification and details of the clinical isolates used in this study

ACTS code (code)	o*mpA* genotype	Clinical outcome
600 (1)	K	Repeat infection, first detection
600 (13)	K	Repeat infection, second detection
620 (1)	D	No repeat infection
628 (1)	E	No repeat infection
649 (1)	K	No repeat infection, genotypically close to 600 (1)

### Infectivity and determination of viable progeny

McCoy B, MCF-7, CACO-2, HeLa, SiHa and ARPE-19 cells were cultured in 96-well plates, in triplicate for each condition and experimental analysis conducted. Cells were infected with each of the five clinical isolates and type strain D/UW-3/Cx at an m.o.i. of 0.5. Cultures were immediately centrifuged at 500 ***g*** and 37 °C for 30 min, then incubated under standard conditions (37 °C, 5 % CO_2_). At 4 h post-infection (h PI), the infectious medium in each well was replaced with complete DMEM supplemented with 1 µg ml^−1^ cycloheximide before further incubation. To determine the infectivity of the isolates in each cell line, cultures were fixed with 100 % methanol at 30 h PI for evaluation by immunofluorescence and microscopy. Infectious progeny counts (inclusion-forming units, IFU) of each isolate was determined from cultures harvested at 44 and 54 h of infection. Inclusion-forming units were determined by serially diluting stocks from each of the harvested cultures onto fresh cultures of McCoy B cells, which were fixed at 30 h PI for evaluation by immunocytochemistry.

### *ompA* genotyping

DNA was extracted from the original swab samples collected (as per the ACTS protocol) and swirled in 500 µl of PBS solution. A 200 µl aliquot of the swab/PBS homogenate was extracted using the MagNA Pure 96 (Roche Applied Science) automated system, according to the manufacturer’s instructions and utilizing the MagNA Pure 96 DNA and Viral NA Small Volume Kit, and eluted in 100 µl in MagNA Pure 96 elution buffer [11].

All *C. trachomatis* genotype determinations utilized a 5 µl aliquot of PBS swab homogenate elution, utilizing a series of quantitative PCR (qPCR) amplification assays targeting the *ompA* gene of *C. trachomatis* as described previously [37].

### Penicillin and iron deprivation persistence models

Persistence models were conducted in MCF-7 cells. In total, 2.5×10^4^ MCF-7 cell monolayers cultured for 24 h were infected at an m.o.i. of 0.8 (slighly higher than the previous cultures due to expected loss of some organisms in the persistence model). Cultures were immediately centrifuged at 500 ***g*** and 37 °C for 30 min, then incubated under standard conditions (37 °C, 5 % CO_2_, 95 % air). At 4 h PI, the infectious medium in each well was replaced with complete DMEM supplemented with 1 µg ml^−1^ cycloheximide as well as 0, 0.02, 0.05 or 1.0 U ml^−1^ benzylpenicillin (Pen G). 2,2′-Bipyridyl (Bpdl) was used in accordance with the methods previously outlined by Thomson and Carabeo (doses of 400 µM) [38]. Cultures were incubated until 44 h PI, when they were harvested and stocked in SPG for analysis of inclusion-forming units, or washed and media replaced with fresh media without the penicillin or supplemented with FeCl_3_ until 96 or 110 h PI of culture and stocked in SPG. The infectious yield of all cultures at each of the time points was determined by infecting serial dilutions of stored stocks into monolayers of McCoy B cells using the standard protocol, and cultures were fixed with methanol at approximately 30 h PI, before immunocytochemistry and visualization by fluorescence microscopy. Cultures for infectivity yields were routinely conducted in 96- or 48-well plates, and replicate cultures were conducted in 24-well plates on top of 1.5 mm coverslips for the cases where fixing, immunocytochemistry and imaging by microscopy to eludicate phenotype were part of the experiment. Azithromycin treatment was conducted to determine each isolate's MIC using the methodology previously outlined [39]; each isolate's MIC dose was then used to treat the isolate during and in the absence of the persistence inducing conditions to evaluate if the isolate’s capacity to survive the antibiotic treatment at the MIC changed by being in persistence.

### Immunofluorescence microscopy

Cultures were fixed with methanol, permeabilized with triton X-100 (0.5%) in Dulbecco’s PBS (dPBS) and blocked with 1 % BSA in dPBS overnight. Once blocked, cultures were then further incubated with dPBS containing 4′,6-diamino-2-phenyindole (DAPI; 1:40 000) and rabbit sera (1:500) containing antibodies raised against the *C. trachomatis* high-temperature requirement A (HtrA) protein for 1 h at room temperature (antibody and protocol as previously described [40, 41]). Following this, cultures were washed four times with dPBS containing 0.2 % tween 20, and then incubated with dPBS containing an Alexa Fluor 488-conjugated goat anti-rabbit antibody (1:600; Thermo Fisher Scientific) for 45 min at room temperature. The stained cultures were then washed five times more with the dPBS tween solution. Cultures on 13 mm (No. 1.5) coverslips were stained and labelled using the same process, with the addition of a mouse anti-α-tubulin (1:500; Thermo Fisher Scientific) in the first antibody incubation, and an Alexa Fluor 546-conjugated goat anti-mouse antibody (1:600; Thermo Fisher Scientific) in the second incubation. Stained coverslips were mounted to clear glass slides using *n*-propyl gallate (NPG) and sealed using clear varnish around the edge of the coverslip. Stained and labelled plate cultures were visualized and imaged using a Nikon Eclipse Ti-S fluorescence microscope or GE InCell 3000 high-throughput fluorescence microscope. Coverslips were visualized and imaged using a Nikon Eclipse Ti-E confocal microscope.

### Data analysis and graphing

Raw data were compiled using Microsoft Excel 2010 before being transferred into GraphPad Prism version 8.0.0 for Windows for statistical analysis and graphing, with each value and any applicable statistical testing described in the respective figure legends.

## Results

### Clinical isolates differed in infectivity and growth more profoundly than the differences caused by the cell lines

**Fig. 1.**
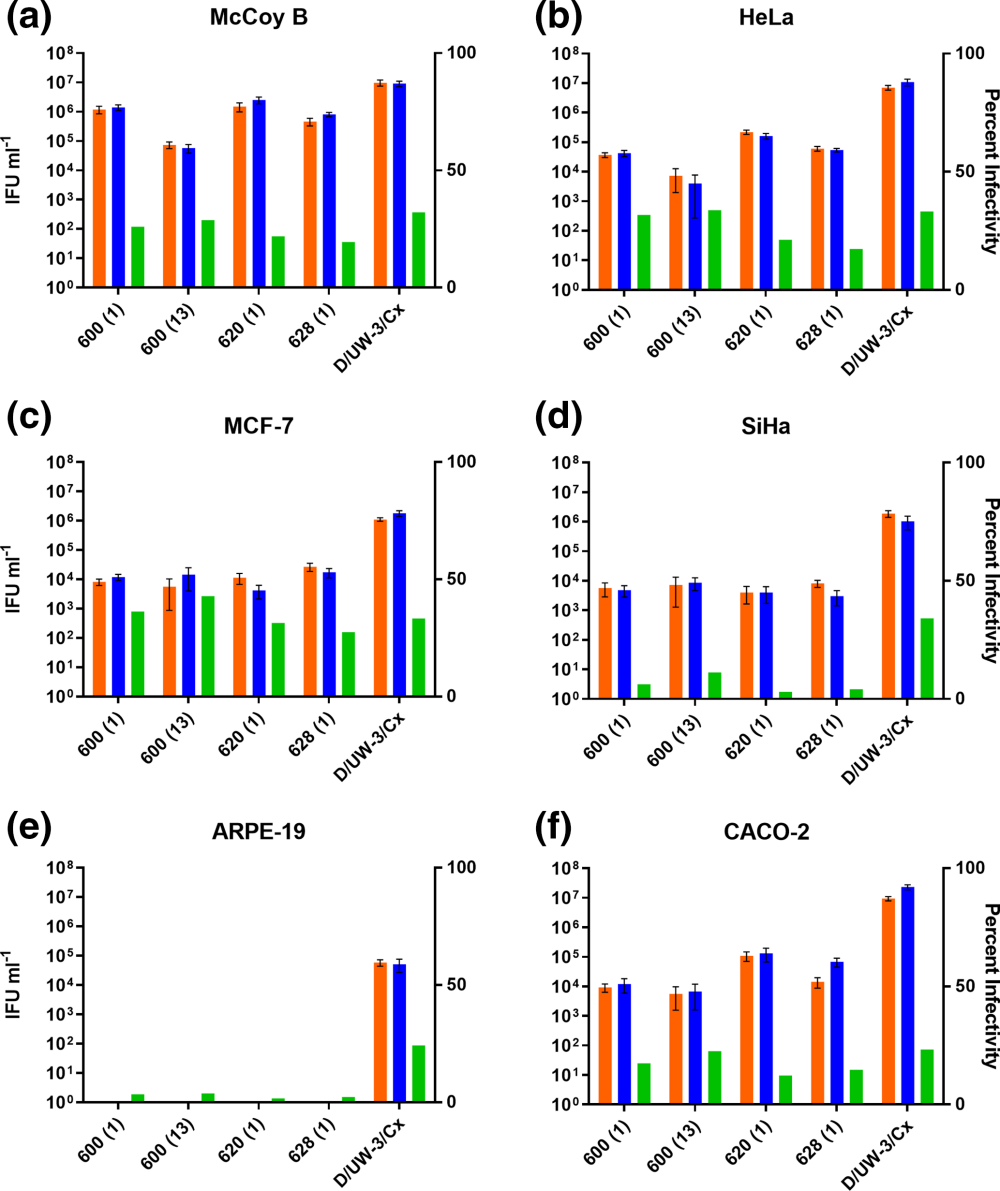
Infectivity and infectious progeny yield in different cell types for the clinical isolates. The figure shows the inclusion forming units (IFU) yielded (left *y* axis) from each isolate (orange indicates yields at 44 h PI and blue indicates yields at 54 h PI) and the percentage infectivity at 30 h PI (right *y* axis, green bar). Each cell line analysed includes (a) McCoy B, (b) Hela, (c) MCF-7, (d) SiHa, (e) APRE-19 and (f) CACO2. The bars for IFU ml^−1^ include a total of *n*=27 representative images to determine the mean of a minimum of *n*=3 separate experimental replicates, shown with standard deviation. The bars for percentage infectivity are representative of *n*=9 experimental replicates. The data are shown from a single experiment, and are consistent with other similar experiments.

Six cell lines were selected, to compare how susceptible they were to infection by four of the clinical isolates, and the type strain D/UW-3/Cx (Fig. 1). The human mammary epithelial cell line (MCF-7) was found to result in the highest cell infection levels after exposure to the chlamydial strains for each of the four clinical isolates, with 35–40 % of all counted cells becoming infected. Both the retinal cell line (ARPE-19) and cervical carcinoma cell line (SiHa) were observed to have the lowest percentage of infectivity (5–10 %) for all four clinical isolates, while the type strain was observed to still have produced inclusions in 25–30 % in both these lines. The proposed 0.5 infectivity or m.o.i. was calculated from an IFU ml^−1^ yield in McCoy B cells. While the type strain showed a similar level of infectivity across the McCoy B, MCF-7, HeLa and SiHa lines, it did show slightly lower levels in CACO-2 and ARPE-19. The same six cell lines used for the infectivity assay were infected and harvested at two time points, to determine the number of infectious progeny that had been produced during the developmental period. All four isolates produced the most infectious progeny (IFU ml^−1^) in the McCoy B cell line (Fig. 1). For example, clinical isolate 600 (1) grown in McCoy B cells yielded 1.2×10^6^ IFU ml^−1^ at 44 h PI, while in HeLa cells, it yielded 3.7×10^4^ IFU ml^−1^. Similarly, the type strain produced higher levels of progeny in the McCoy B cells (9.8×10^6^), in CACO-2 (9.4×10^6^) and HeLa cells (7.1×10^6^), compared to MCF-7 (1.1×10^6^) and SiHa (1.9×10^6^) cells, and ~100-fold fewer in ARPE-19 cells (5.9×10^4^). The ARPE-19 cell line was also shown to produce the least number of infectious progeny for clinical isolates, with all four showing no detectable progeny. Less variation in the infectivity and infectious progeny production from each of the clinical isolates was observed in the SiHa cell lines compared to the other cell lines (Fig. 1).

### Clinical isolates show similar susceptibilities to conditions of iron deprivation at lower doses than type strain D/UW-3/Cx

**Fig. 2.**
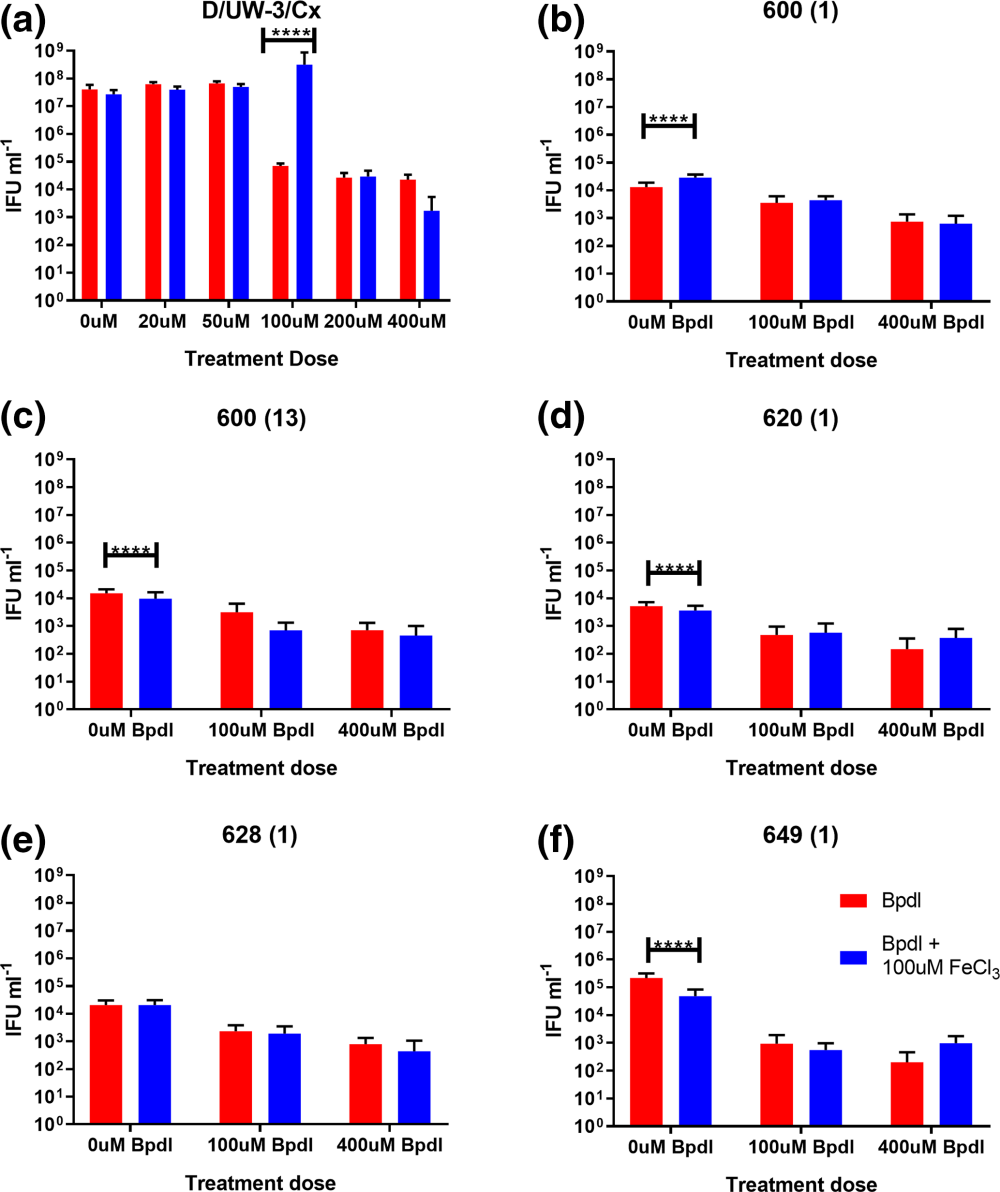
Iron deprivation-related persistence and recovery of the clinical isolates. The graphs show the infectious progeny produced by type strain D/UW-3/Cx (**a**) and five clinical isolates [(b): 600 (1); (c): 600 (13); (d) 1-020 (1); (e) 628 (1); (f) 649 (1)] in MCF-7 cells treated with Bpdl±100 µM iron as FeCl_3_ for recovery. Each bar represents the mean of three experimental replicates analysed using *n*=10 separate fields of view from *n*=3 experimental replicates with error bars showing the standard error of the mean (sem). Data were analysed by a three-way ANOVA using GraphPad Prism version 8.0.0 for Windows. Significant differences are indicated by ****P<0.0001. The data are shown from a single experiment, and are consistent with other similar experiments.

To more closely examine if persistence phenotypes were impacted by *ompA* genotypes, and the known outcomes in participants in the ACTS trial, an additional isolate was included in these persistence experiments to have a close genotype to the isolate from a participant who experienced repeat infection. Five clinical isolates and the type strain were cultivated in MCF-7 cells, treated with 100, 200 (D/UW-3/Cx only) and 400 µM bipyridal (Bpdl)±100 µM iron as FeCl_3_, with infectious progeny determined at 96 h PI. As seen in Fig. 2a, the type strain was able to recover from up to 100 µM Bpdl (7.1×10^4^ IFU ml^−1^ before recovery) with the supplementation of FeCl_3_ into culture (3.1×10^8^ IFU ml^−1^ after recovery). The five clinical isolates showed lower recovery, even from the lowest dose of Bpdl, as seen in Fig. 2. For example (Fig. 2c), at the 100 µM dose of Bpdl with and without recovery, 600(13) yielded 3.1×10^3^ and 7.0×10^2^ IFU ml^−1^ (near the limit of detection for this assay) respectively. All five isolates produced infectious progeny in a manner that was dose-dependent, with slightly lower levels of infectious progeny present in cultures treated with 400 µM than those treated with 100 µM.

### Treatment with azithromycin during iron deprivation-induced persistence decreases the number of recoverable infectious progeny among clinical isolates

**Fig. 3.**
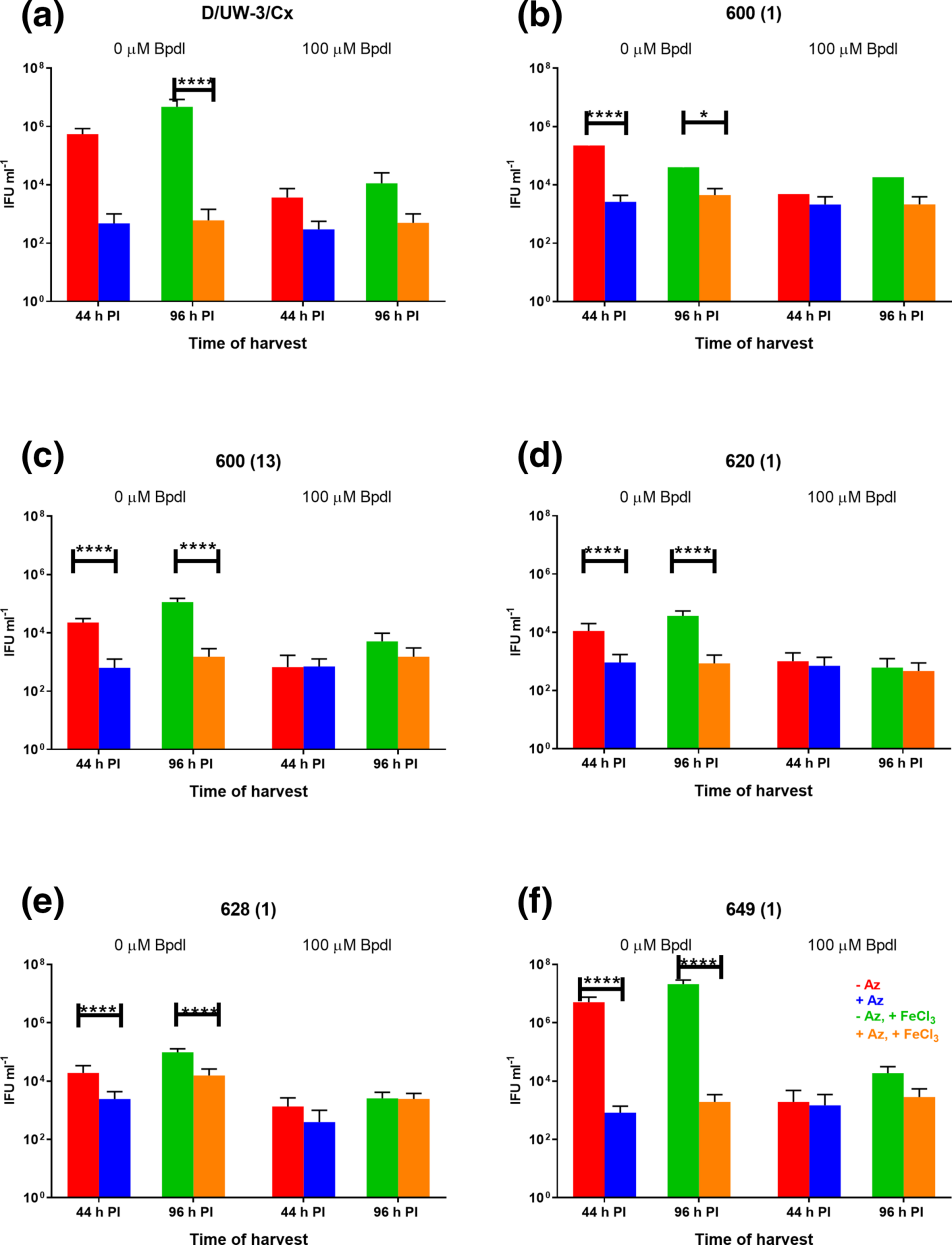
Iron deprivation-induced persistence and recovery of the chlamydial isolates. Infectious progeny (IFU, *y* axis) produced by the type strain D/UW-3/Cx (**a**) and five clinical isolates [(b): 600(1); (c) 600(13); (d) 620 (1); (e) 628(1); (f) 649(1)] in MCF-7 cells treated with Bpdl, Az±100 µM iron as FeCl_3_ for recovery. Azithromycin dose was the MIC for the strain; 600 (1): 0.032 µg ml^−1^, 600 (13): 0.125 µg ml^−1^, 620 : 0.032 µg ml^−1^, 628 : 0.064 µg ml^−1^; 649 : 0.064 µg ml^−1^, and D/UW-3/Cx: 0.064 µg ml^−1^. Each bar represents the mean of three experimental replicates analysed using *n*=10 separate fields of view from *n*=3 subsequent infectivity (passage) wells with error bars showing sem. Data were analysed by a three-way ANOVA using GraphPad Prism version 8.0.0 for Windows. Significant differences are indicated by ****P<0.0001 and *P=0.0138. The data are shown from a single experiment, and are consistent with other similar experiments.

**Fig. 4.**
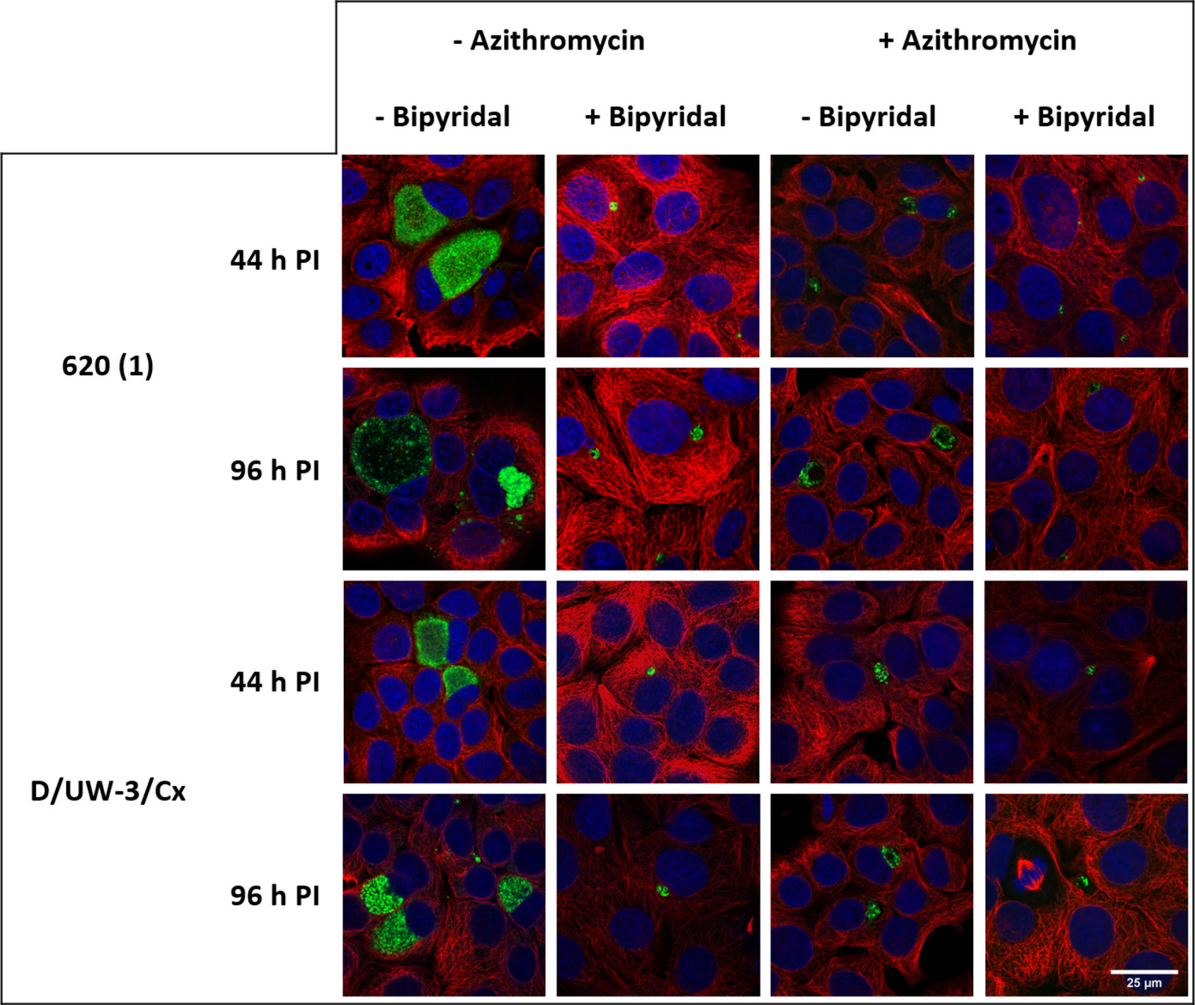
Confocal microscopy of *Chlamydia* cultures during iron-deprivation persistence. MCF7 cells infected with 620 (1) (top two rows) or type strain D/UW-3/Cx (bottom two rows) were fixed and visualized using immunofluorescence. Active and persistent inclusions containing chlamydial HtrA appear as green, while the host alpha-tubulin appears as red and the host cell nucleus as blue. These images are representatives of observations for each experimental condition.

Each of the five clinical isolates and the type strain were cultured in MCF-7 cells treated with Bpdl, subsequently treated with azithromycin (Az), and then recovered with supplementation of iron. The azithromycin dose used was the MIC that had been determined for each isolate. The MICs were as follows: 600 (1): 0.032 µg ml^−1^, 600 (13): 0.125 µg ml^−1^, 620: 0.032 µg ml^−1^, 628 : 0.064 µg ml^−1^; 649 : 0.064 µg ml^−1^, and D/UW-3/Cx: 0.064 µg ml^−1^. All strains showed a significant decrease in infectious progeny at 44 h PI when treated with azithromycin, Bpdl or both (Fig. 3, *P*<0001). Compared to the untreated control at 44 h PI (5.4×10^5^ IFU ml^−1^), D/UW-3/Cx showed an impaired ability to recover from the effects of Bpdl when also treated with azithromycin (2.9×10^4^ IFU ml^−1^), even with iron supplementation (5.0×10^2^ IFU ml^−1^). Compared to their respective untreated controls at 44 h PI (Fig. 3), all five clinical isolates were found to have only slight differences in their resulting infectious yield when treated with the combinations of azithromycin, Bpdl and FeCl_3_. For example, the untreated control 600 (13) culture produced 2.2×10^4^ compared to only 1.5×10^3^ IFU ml^−1^ after treatment and recovery. Analysis of the cultures by confocal microscopy showed morphologies consistent with persistence, as inclusions visible were consistent with persistence after treatment with penicillin; regular development occurred after allowing for recovery from the drug (Fig. 4, representative images from some isolates).

### Clinical isolates entered persistence at lower doses of penicillin compared to the type strain

**Fig. 5.**
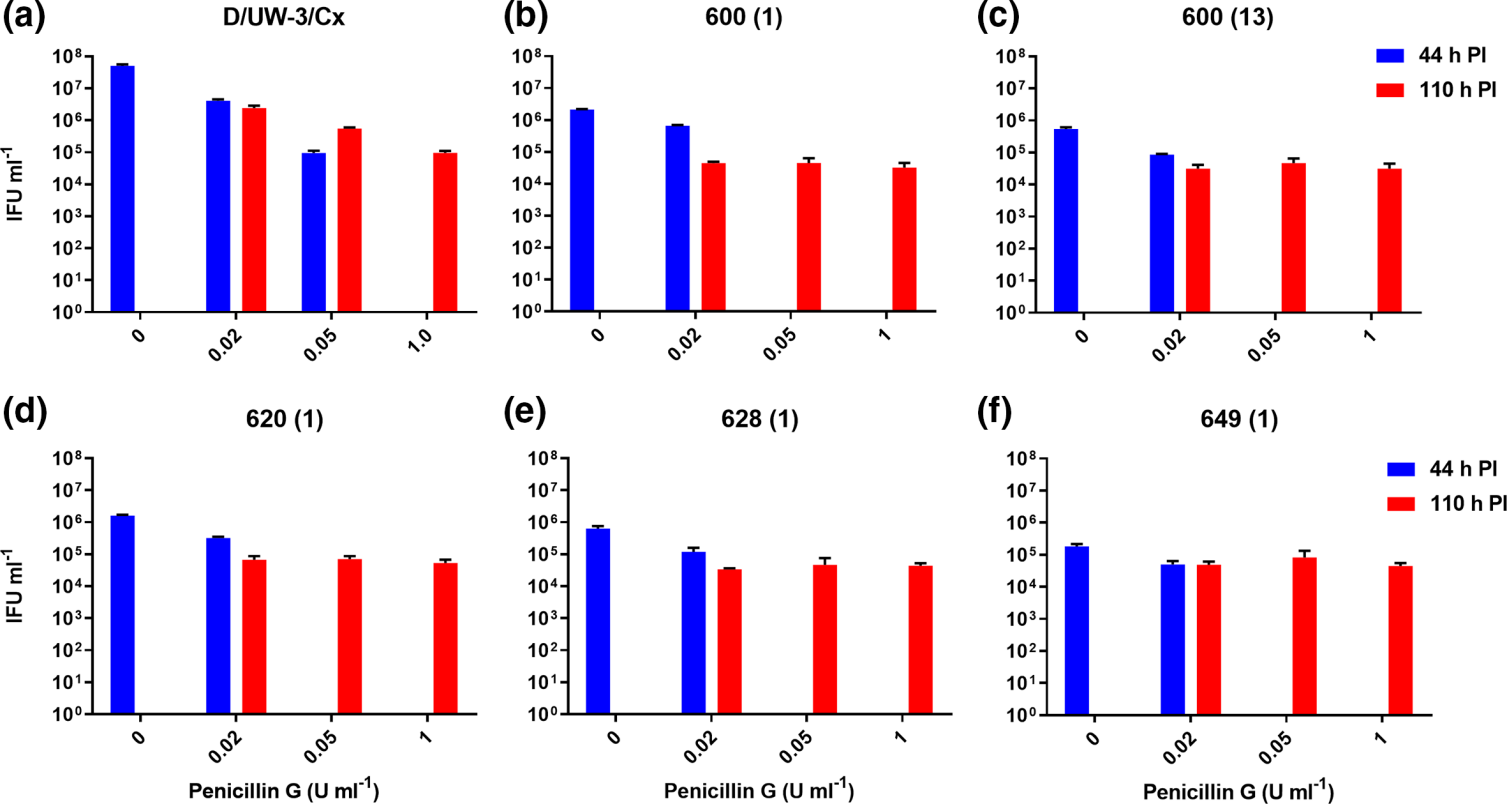
Enumeration of infectious progeny from penicillin persistence experiments. IFUs from culture harvested at 44 and 110 h PI are shown on the graph(s). The IFU ml^–1^ (*y* axis, log scale), and dose of penicillin (*x* axis), and recoverable IFU ml^–1^ at 44 h PI (blue bars, during persistence), and 110 h PI (red bars, recovery). Data shown represent the mean value of *n*=3 separate experimental replicates wells with *n*=10 images analysed from each of *n*=3 wells, with error bars representative of the sem. The data are shown from a single experiment and are consistent with other similar experiments. These data were used to select the dose of penicillin G for the next experiments for each isolate and as such were not used for statistical analysis.

**Fig. 6.**
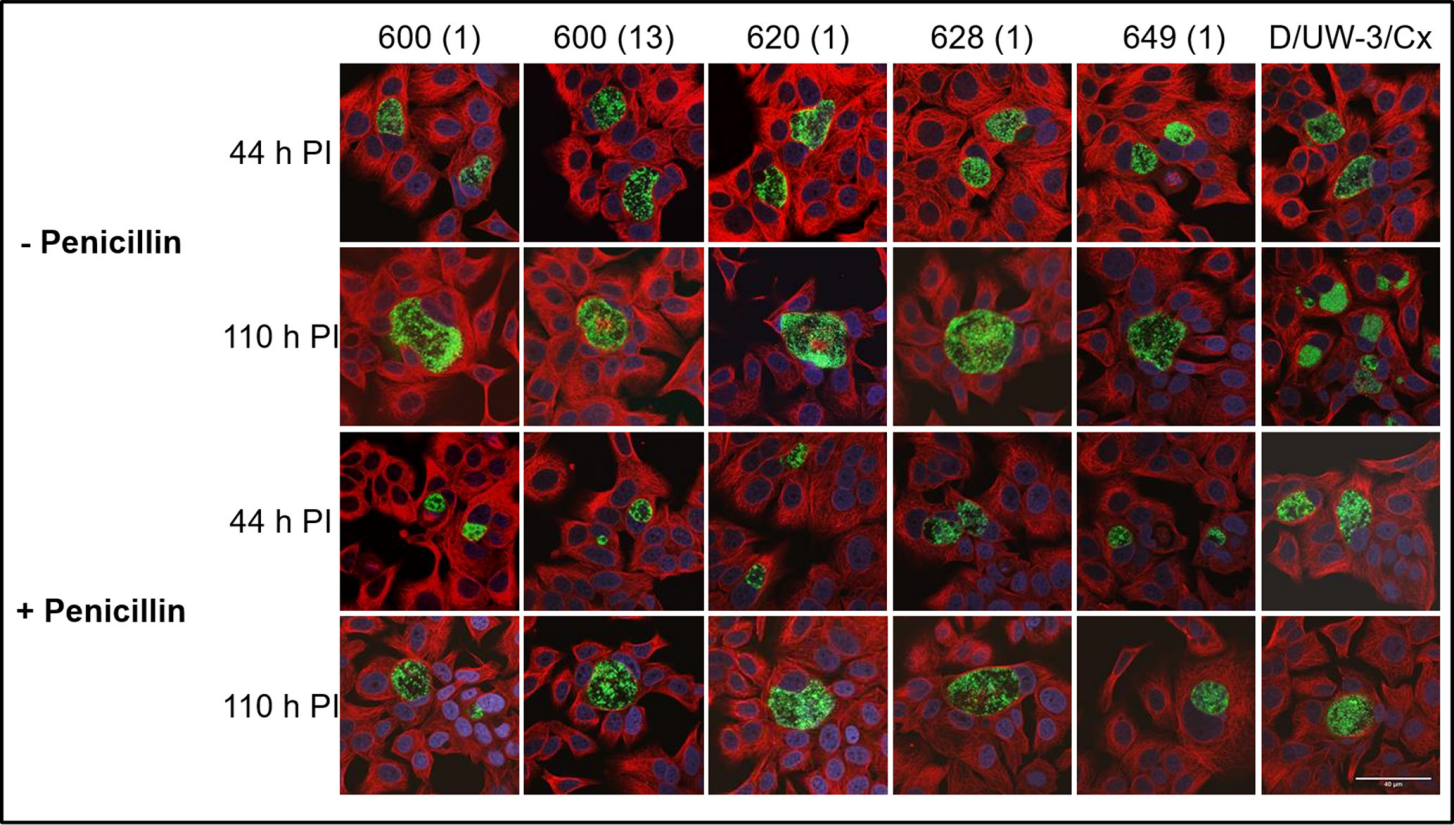
Confocal microscopy of the chlamydial cultures in the presence and absence of penicillin and post-recovery. Active and persistent inclusions containing chlamydial HtrA appear as green, while the host alpha-tubulin appears as red and the host cell nucleus as blue. These images are representatives of observations for each experimental condition.

Several doses of penicillin were used to induce persistence in each strain, which was confirmed by measuring viability before and after recovery from the drug. The data shown in Fig. 5 demonstrate that compared to the untreated controls at 44 h PI, the addition of 0.02 U ml^−1^ penicillin slightly impacted the development of each clinical isolate, while the 0.05 and 1.0 U ml^−1^ doses had rendered them non-cultivable. Conversely, only the 1.0 U ml^−1^ dose caused the type strain to enter persistence, with far less pronounced effects than the clinical isolates prevalent at the lower doses. Isolates 600 (1) and 600 (13) produced 2.1×10^6^ and 5.5×10^5^ IFU ml^−1^ in their respective untreated cultures, compared to 6.6×10^5^ and 8.7×10^4^ IFU ml^−1^ in the cultures treated with the lowest dose of penicillin. The type strain exhibited only slightly impacted growth at both the lower doses (5.0×10^7^ IFU ml^−1^ untreated versus 4.1×10^6^ and 9.4×10^4^ IFU ml^−1^ at 0.02 and 0.05 U ml^−1^, respectively), and only became non-cultivable at the 1.0 U ml^−1^ dose. Each of the five clinical isolates and the type strain were able to produce detectable (after recovery) infectious progeny by 110 h PI, all to a similar level, even after treatment with 1 U ml^−1^ of penicillin. Confocal microscopy examination of the morphology of the cultures confirmed the presence of visible forms consistent with persistent or recovered morphology (Fig. 6). Specifically, at 44 and 110 h PI and in the absence of penicillin, each of the five clinical isolates and the type strain showed typical morphologies consistent with regular development. At the same time point, cultures treated with penicillin showed significantly smaller inclusions with enlarged particles inside, morphologies consistent with persistence. Imaging of the cultures at 110 h PI (66 h after the removal of penicillin from culture) showed inclusions typical of regular inclusions.

### Clinical isolates treated with azithromycin during penicillin persistence showed a dose-dependent decrease in recoverable infectious progeny

**Fig. 7.**
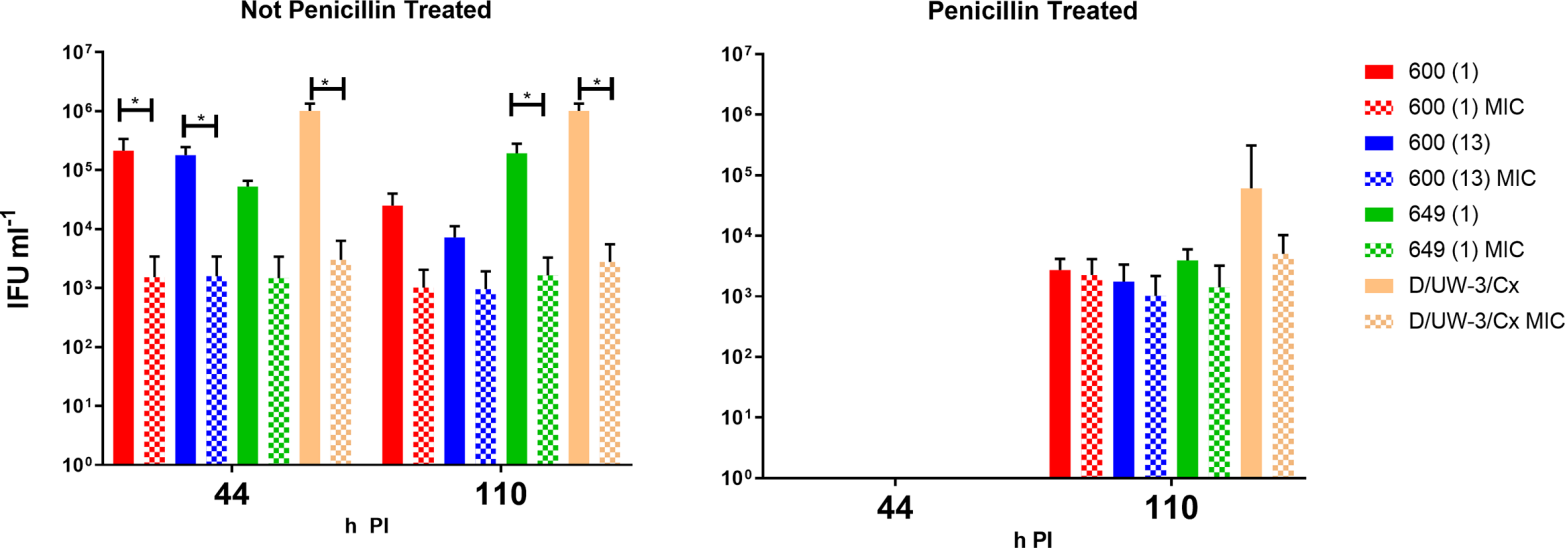
Impact of azithromycin treatment during penicillin-induced persistence. The graphs show the IFU ml^–1^ of each clinical isolate and type strain D/UW-3/Cx in the presence of azithromycin (at the MIC for that strain), with and without pretreatment and recovery from benzylpenicillin. Azithromycin dose; 600 (1): 0.032 µg ml^−1^, 600 (13): 0.125 µg ml^−1^, 620 :0.032 µg ml^−1^, 628 : 0.064 µg ml^−1^; 649 : 0.064 µg ml^−1^, and D/UW-3/Cx: 0.064 µg ml^−1^. The *x* axis shows each isolate grouped at both time points on the left- and right-hand sides respectively and the *y* axis shows infectivity of the cultures as IFU ml^−1^. Data shown represent the mean value of *n*=3 separate experimental replicate wells with an average of *n*=10 images analysed to input the result for each of *n*=3 wells, with error bars representative of the sem. The data are shown from a single experiment, but are consistent with other similar experiments. The *P* values for the significant different outcomes for the isolates are as follows (left to right): 0.043, 0.099, 0.066, 0.027, 0.0066.

To assess whether clinical isolates had altered susceptibilities to azithromycin during persistence, three clinical isolates and D/UW-3/Cx were cultured in MCF-7 cells and treated with both penicillin and azithromycin. Persistence was induced using 0.05 U ml^−1^ of penicillin for the three clinical isolates and 1.0 U ml^−1^ for the type strain. At 44 h PI, Fig. 7 shows a complete loss of infectious progeny for the penicillin-treated cultures, which was recoverable (albeit reduced) by 110 h PI. The infectious progeny of the four strains at 44 h PI was also observed to decrease by up to 100-fold when treated with azithromycin alone. In the type strain, this effect was also seen, whereby the recoverable infectious progeny decreased in the presence of azithromycin only, with a complete loss of viability at 44 h PI when both azithromycin and penicillin were added. However, no statistical differences were apparent for any isolates treated with azithromycin during penicillin persistence. It appeared that the recovery of the clinical isolates from persistence was further reduced when treated with azithromycin at the MIC, with two of the isolates [600 (13) and 649] appearing to be more impacted by the combination treatment.

## Discussion

The susceptibility and growth permissiveness of different cell lines to infection by *C. trachomatis* enables examination of the host–pathogen relationship [42–45]. Such studies frequently find differences between strains in their ability to enter the host cell, and complete their developmental cycle [46]. The infectivity of the clinical isolates in the present study was observed to be highest in the MCF-7 cell line, despite the long-standing practice throughout the field of using McCoy or HeLa cells for cultivation and isolation of *C. trachomatis *, especially from clinical samples [47–49]. However, infectious yields were highest in the McCoy B cell line for each of the strains tested. The ARPE-19 cell line was observed to have a very low susceptibility to infection by, and permissiveness of growth to, all strains except D/UW-3/Cx, yet was nonetheless able to be infected by each of the strains, even though it is not an epithelial cell type. The inability to detect progeny from this cell line for the clinical isolates may reflect the low yields being below detection of the assay, or that the clinical isolates in these cells are unable to form infectious elementary bodies. Overall, these findings reinforce that there are phenotypic differences between type strains and clinical isolates that probably reflect the adaptation of D/UW-3/Cx to growth *in vitro* [50].

Although it has been shown that type strains of *C. trachomatis* have varying levels of susceptibility to the *in vitro* effects of IFN-γ, fewer studies have examined how different strains respond to penicillin or iron deprivation [51]. In this study, we aimed to determine how clinical isolates respond to such conditions. Both were selected on the basis that penicillin has been used widely as a chlamydial persistence model, so our findings can be interpreted in light of other studies [36, 52–54]; iron deprivation is potentially a more clinically relevant model when considering the physiology of the female reproductive tract [55–57]. Penicillin is known to induce persistence by interacting with chlamydial penicillin-binding proteins (PBPs), while host cell-derived iron is essential for chlamydial development. Both points combined raise the possibility that genetic variation among infecting strains could result in differing thresholds at which they divert from the regular developmental cycle into persistence.

To assess the susceptibility of each strain to penicillin, they were treated with a dose range in MCF-7 cells. None of the clinical strains showed any notable difference in their levels of susceptibility to penicillin persistence, with all entering a viable but non-cultivable state at 0.05 U ml^−1^ of the antibiotic. In contrast, the type strain D/UW-3/Cx remained cultivable up to the maximum dose used, which was 1 U ml^−1^. A recent study into the effects of beta-lactam antibiotics on *C. trachomatis* showed that type strain E/UW-3/Cx entered persistence at 0.02 U ml^−1^ of benzylpenicillin [36]. Although using different host cells, this suggests there may be different susceptibilities among type strains.

Similarly, there were no large observed differences in the responses of clinical isolates to the effects of iron deprivation. In a recent review, Pokorzynski and colleagues postulated a complex system by which *C. trachomatis* may be able to both passively and actively acquire ferrous and ferric iron from within the host cytoplasm by modulating the host's own iron trafficking pathways [58]. As with other instances of chlamydial persistence, it is possible that strain differences may in turn result in slight differences in the proteins that conduct these functions.

The effects of azithromycin were tested on a selection of clinical isolates during both active and persistent development. We observed no differences in the susceptibilities of active and persistent infections, in any of the clinical strains tested. Previous findings by the Caldwell group demonstrate that during IFN-γ-mediated persistence, a serovar D type strain of *C. trachomatis* is significantly more susceptible to azithromycin [35]. However, Wyrick and Knight have shown that a serovar E type strain is less susceptible to the same antibiotic during penicillin-mediated persistence [52]. This reinforces previous findings that different inducers of persistence produce different phenotypes [59], which probably reflects the physiological stress the inducer places upon *C. trachomatis *.

Collectively these findings show that clinical isolates respond to the effects of penicillin, iron deprivation and azithromycin in a similar but more pronounced way than type strain D/UW-3/Cx. This is important because these are recent clinical isolates, indicating that persistence may occur more frequently and with lower thresholds *in vivo*. Here we demonstrated that clinical isolates showed subtle variation in thresholds for persistence that may be more distinct and impactful in the complex *in vivo* environment. These variations may translate into phenotypes *in vivo* that could relate to heterogenic survival of antibiotic treatment, or different capacities to survive in different tissue niches (e.g. rectal compared to urogenital) that could be relevant for future investigation of chlamydial variation.
